# Personal customizing exercise with a wearable measurement and control unit

**DOI:** 10.1186/1743-0003-2-14

**Published:** 2005-06-28

**Authors:** Zhihui Wang, Tohru Kiryu, Naoki Tamura

**Affiliations:** 1 Graduate School of Science and Technology, Niigata University, 8050 Ikarashi-2nocho, Niigata 950-2181, Japan

**Keywords:** wearable unit, personally customized workload control, information technology, biosignal, cycle ergometer, appropriate exercise level

## Abstract

**Background:**

Recently, wearable technology has been used in various health-related fields to develop advanced monitoring solutions. However, the monitoring function alone cannot meet all the requirements of customizing machine-based exercise on an individual basis by relying on biosignal-based controls. We propose a new wearable unit design equipped with measurement and control functions to support the customization process.

**Methods:**

The wearable unit can measure the heart rate and electromyogram signals during exercise performance and output workload control commands to the exercise machines. The workload is continuously tracked with exercise programs set according to personally customized workload patterns and estimation results from the measured biosignals by a fuzzy control method. Exercise programs are adapted by relying on a computer workstation, which communicates with the wearable unit via wireless connections. A prototype of the wearable unit was tested together with an Internet-based cycle ergometer system to demonstrate that it is possible to customize exercise on an individual basis.

**Results:**

We tested the wearable unit in nine people to assess its suitability to control cycle ergometer exercise. The results confirmed that the unit could successfully control the ergometer workload and continuously support gradual changes in physical activities.

**Conclusion:**

The design of wearable units equipped with measurement and control functions is an important step towards establishing a convenient and continuously supported wellness environment.

## Introduction

In rehabilitation engineering and health promotion, personally customized control of machine-based exercise should be introduced to reflect gradual changes in individual physical work capacity [1]. Biosignal-based workload control systems show great promise as an effective approach to regulate exercise levels [2-4]. Generally, exercise levels are adjusted manually for specific exercise machines, in specific places, typically only by physicians with expertise in sports medicine [5-7]. We have developed an Internet-based cycle ergometer exercise system, aimed at providing a personally customized workload control any time in convenient locations [8,9]. In this system, exercise resources including exercise programs and workload patterns are distributed over the Internet and dynamically integrated on the cycle ergometer. Workload patterns provided by clinicians are computer files defining the time-course of the exercise to meet individual fitness levels and ability. In practical applications, we prepared and set-up measurement equipment, such as computers, amplifiers, and A/D converters, for individual machines. Unlike these conventional systems, significant advances in wearable technology allow us to continuously assess human biometrics more conveniently. Thus, a wearable unit equipped with measurement and control functions can be used on various machines. That is, by setting up one unit, users can perform biosignal-based exercises at a consistent pace, even on a variety of exercise machines. Accordingly, wearable units have the potential to advance the personal customization process, thereby providing a better exercise routine on an individual basis. A lot of attention has been directed to the investigation of health monitoring services, and various types of wearable unit coordinated monitoring function have been studied [10-14]. Still, there are no wearable units suitable for personally customized machine-based exercise. To implement such units, the workload control function must be embedded into the wearable units, and consequently the units can output control signals to the exercise machines to set the appropriate exercise levels.

Because exercise machines used in gyms/health clubs are configured in very different ways, (e.g., some machines have measurement and control functions, while others do not), most users find it very inconvenient to perform exercise in different places. To provide a personal customizing exercise, we need to measure the biosignals and control the workload without any constraints on machines and locations. Therefore, we separated the measurement and control functions from the exercise machines and incorporated these functions into one wearable unit. This allows the personally customized workload control to be implemented at any convenient place. Another disadvantage of traditional exercise machines is that most of them only provide pre-installed exercise programs with limited variations [15]. This is not cost-efficient because upgrading the exercise programs is very complicated and sometimes impossible. In this case, wearable units equipped with measurement and control functions can be used to loosely couple the exercise machines and programs to easily revise and upgrade conventional exercise programs at end users.

We studied biosignal-based workload control, in which the workload can be adjusted using fuzzy inference to continuously adapt the exercise as a function of heart rate and muscle activity [2]. In this paper, we propose a new design of wearable unit for machined-based exercise. To support the personal customization process, we build the measurement and control functions into a single wearable unit. The unit has several different interfaces for measuring multiple biosignals during exercise and then output control commands to exercise machines. To improve convenience, communications between the exercise machines and the wearable unit are by wireless connections. We developed a prototype of this wearable unit for cycle ergometer exercise and used it as part of an Internet-based exercise system. We examined the wearable unit by recruiting nine volunteers over a two-month period. Our results showed that the wearable unit was effective to handle changes in physical activity while controlling the cycle ergometer and was expected to provide continuously supporting appropriate workload patterns for individuals.

## Methods

To customize exercise protocols on an individual basis, we need timely updates of workload patterns and continuous workload adjustment, based on the analysis of various biosignals, such as the heart rate (HR) and electromyogram (EMG) signals [1]. Wearable units must offer these measurement and control functions. To enable users to exercise regardless of time and place, the unit must be designed to obtain exercise programs and workload patterns via the Internet and to automatically submit the exercise results.

### Wearable Unit Design

Wearable units for machine-based exercise should have interfaces to measure the biosignals. The kind of biosignals required depends on the type of control to be used in exercise programs. We used HR and EMG signals to compute the appropriate exercise levels, according to the idea that gradual changes in physical activity are of interest during an exercise routine. Although exercise programs can be embedded into the wearable unit, they would require a significant amount of the unit's resources, especially if the programs include complicated control methods. Due to the limited processing power and storage capacity available via wearable units, the optimal configuration has wired or wireless communication interfaces to connect to external computers with relatively high performance. If necessary, external computers are utilized for executing exercise programs to provide control parameters. In this case, the wearable unit is a type of middleware, linking the exercise machines to the exercise programs. In addition, like typical designs, the wearable unit needs to have adequate data measurement capacity and transfer speed. Most importantly, the wearable unit should be equipped with an A/D converter and amplifier that operate independently from each exercise machine.

**Figure 1:**
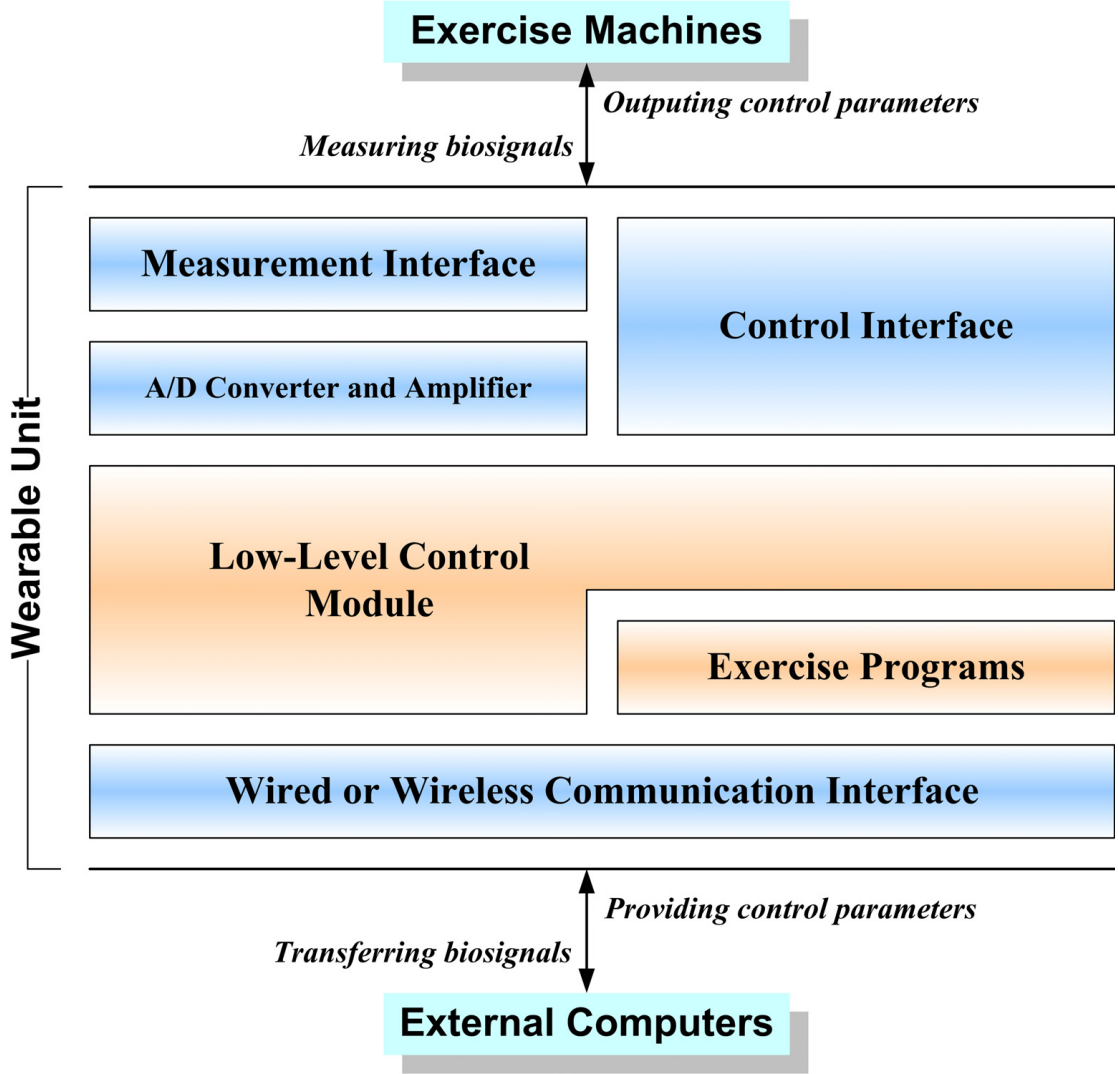
Schematic representation of the design of the wearable unit for machine-based exercise.

Figure 1 presents our overall design of a wearable unit that meets the requirements of the above design considerations. The low-level control module fixed in the unit is responsible for detecting TCP connections, dealing with temporal biosignal data, and generating control commands according to the specifications of the different exercise machines. Note that the exercise programs can reside either on the wearable unit or on an external computer. The decision about which approach to use depends on the complexity of the exercise programs.

### Prototype of a Wearable Unit for Cycle Ergometer Exercise

We developed a prototype of the wearable unit to dynamically control the workload during cycle ergometer exercise. It has a Linux (kernel 2.4) operating system, a 66-MHz-CPU, and 2-MB memory capacity. It also has an on-board 12-bit resolution A/D converter, a 60-dB-gain amplifier, a PCMCIA type slot for a wireless LAN card, and an IP address. Additionally, it features 6 channels for biosignal measurements and a sampling frequency of 5 kHz. At the present development stage, infrared wireless communication is used to acquire HR information from, and output workload control commands to, a cycle ergometer.

Our provided exercise program contains a procedure to calculate the appropriate workload by estimating HR and EMG signals, using a set of predefined fuzzy rules and membership functions [2]. The procedure is time-consuming and requires storage space for the measured data (more than 8-MB for each exercise course). The wearable unit cannot work alone to provide the workload because of its low current capacity. Therefore we used external computers to execute the exercise program and compute the workload. Data transmission between the unit and external computers was implemented using TCP socket communication over wired or wireless connections. At the time of workload control, the unit's built-in low-level control module (Fig. 1) created separate threads to communicate with the external computers and cycle ergometer. Hence, the measurement, control, and data transmission processes were performed individually.

**Figure 2:**
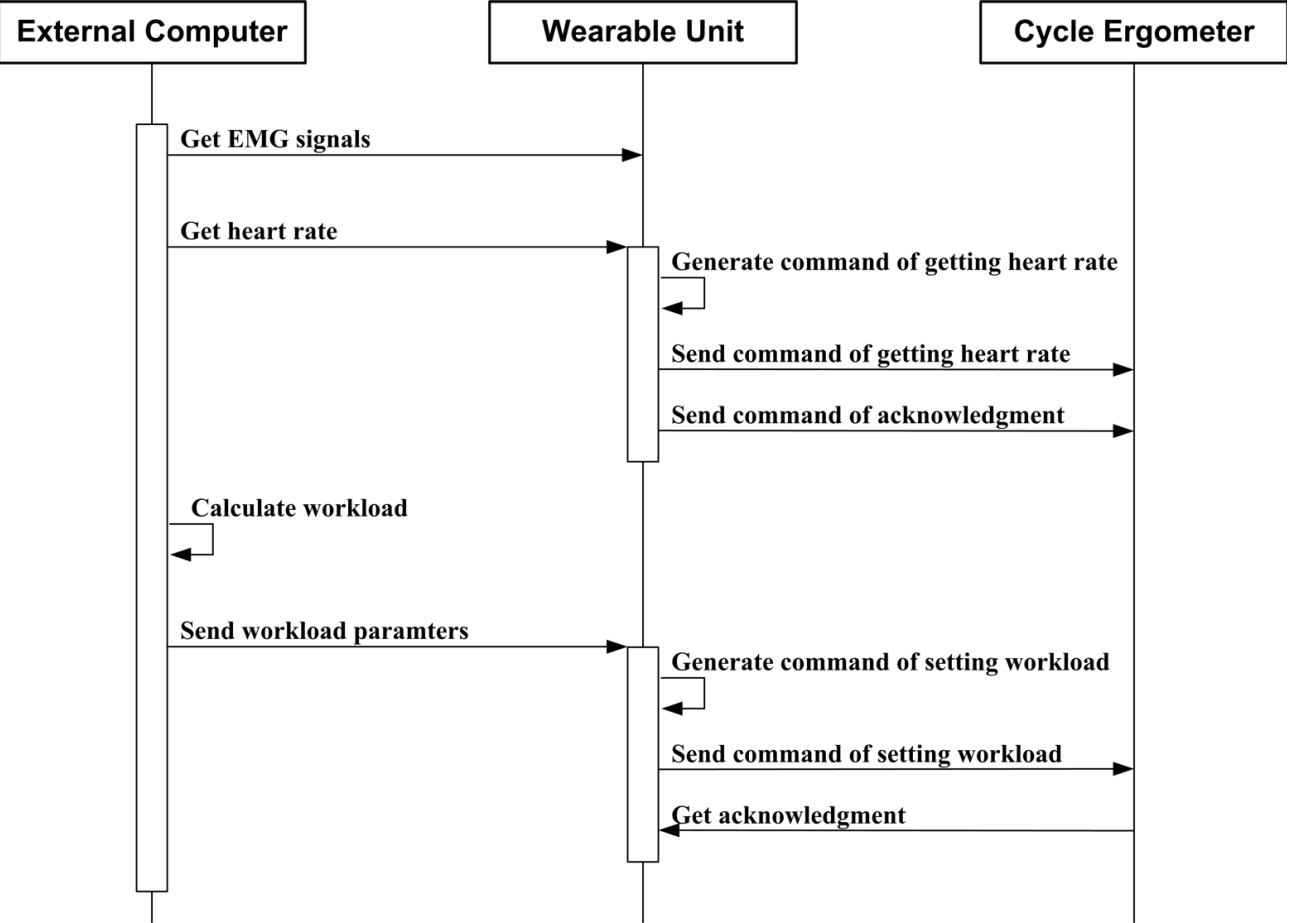
Acquisition-control sequence diagram for controlling the cycle ergometer through the wearable unit with the help of an external computer.

Figure 2 shows an acquisition-control sequence diagram of how the wearable unit works with a cycle ergometer and an external computer. Note that at first, the exercise program residing at the external computer opens a TCP connection to the wearable unit. Through this connection, the program acquires and records the HR and EMG signals, measured by the unit. The external computer calculates the workload parameters and sends them to the unit. When receiving the workload parameters, the unit parses them to generate the corresponding workload setting command, and then submits the command to the cycle ergometer. In addition, the exercise program stores all the measured data on the local disk of the external computer for future design of workload patterns. It is worth to emphasize that the exercise program does not reside on cycle ergometers, but rather on external computers. Thus, we can easily upgrade the program without tampering with cycle ergometers.

### Applying The Wearable Unit to Internet-Based Exercise Systems

We have developed an Internet-based cycle ergometer exercise system [8,9], which is the backbone of support for the wearable unit, in terms of easy access to various exercise resources at any time from any place. The system provides a central server to process client requests and a history database to store the exercise resources. We have also provided a utility to help clinicians design workload patterns [16]. By coordinating the wearable unit with this system, the practicality and convenience of the personal customization process will improve, because the unit will be able to accommodate various types of cycle ergometers, regardless of whether or not they already have embedded measurement and control functions.

**Figure 3:**
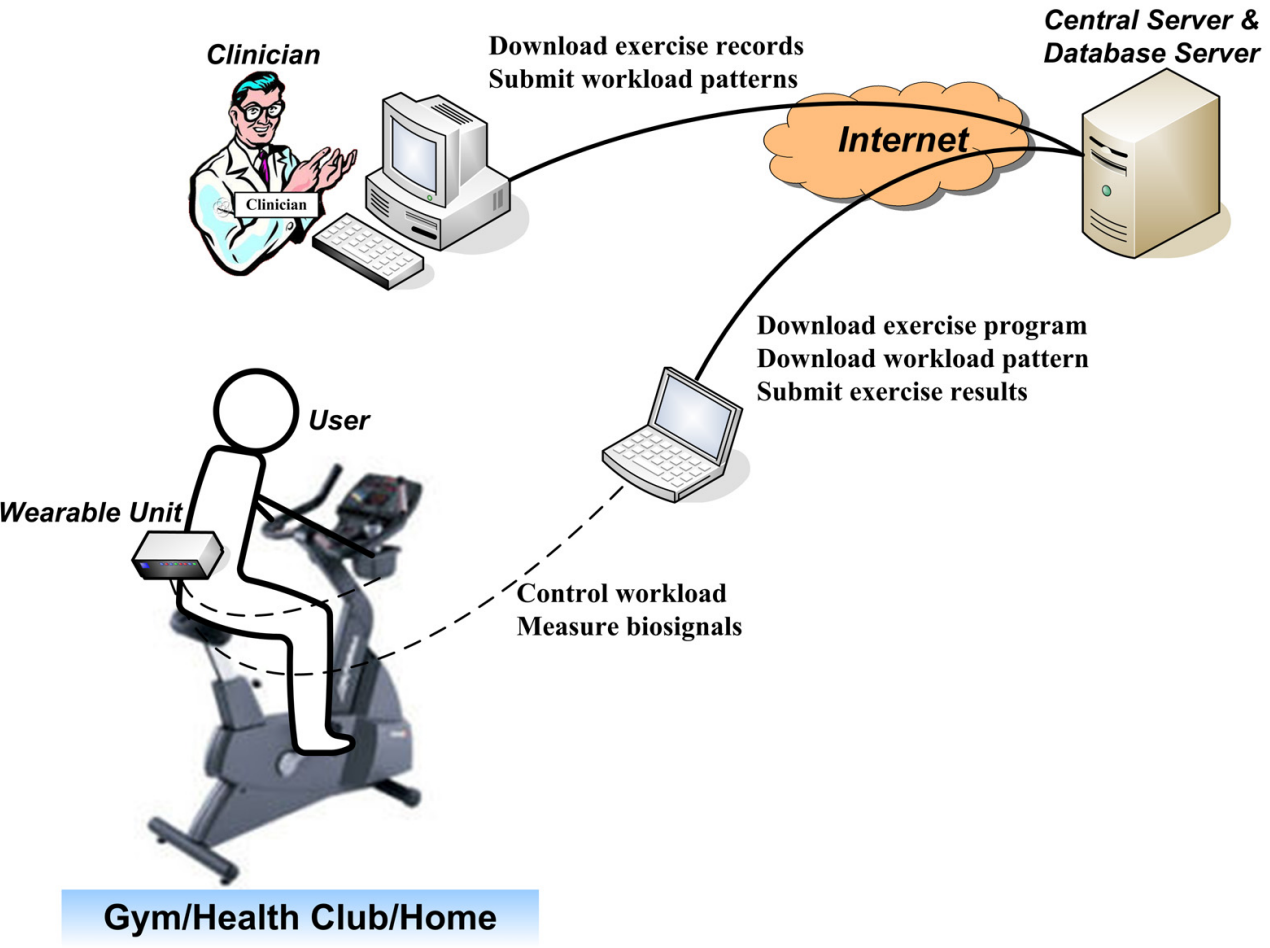
Layout of Internet-based cycle ergometer exercise system. There is an external computer in the exercise location that communicates with the central server. Clinicians can remotely design and send workload patterns, which will be downloaded by the users at the time of exercise.

The proposed exercise system (Fig. 3) is composed of a central server and a database server for both the users and physicians with expertise in sports medicine. Clinicians are responsible for designing appropriate workload patterns, based on a review of the database history, and for remotely uploading the patterns. At the user's location, external computers communicate with the central server to download the exercise program and the latest workload pattern designed by clinicians. The downloaded exercise program continuously transmits the workload parameters to the wearable unit via a wireless connection, and then, the unit sets the workload level on the cycle ergometer. The wearable unit gathers HR and EMG and sends this data to the external computer. The exercise program automatically submits all the exercise results to the central server via the Internet after the exercise session is finished.

## Results

**Figure 4:**
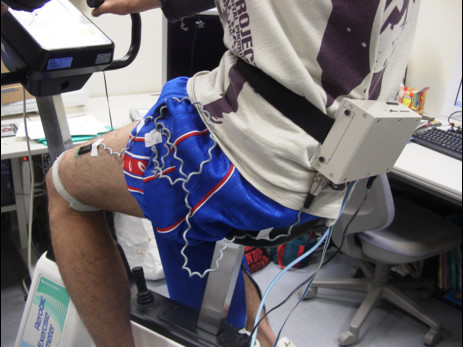
Photograph of the unit being worn during cycle ergometer exercise.

We conducted a set of field experiments with the wearable unit over a two-month period in a hypothetical Internet-based environment, using 100-Base-T Ethernet connections, set up in our laboratory. The purpose was to test the system to personally customize workload control while subjects were using a cycle ergometer and physiological data were gathered using the wearable unit. Figure 4 shows an actual exercise session of a subject wearing the unit around his waist. The design utility [16] was installed in advance on a computer operated by a clinician. The experiments were centered on the Microsoft Windows system (Windows 2000). In addition, subjects and clinicians worked in different places.

Seven male and two female young subjects (21.3 ± 1.7 years old) assisted us in carrying out the experiments. They exercised once or twice a week for 30 minutes at a time. The exercise flow was the same as for our previous study on the personal customizing exercise [1]. At first, all subjects took a *progressively increasing workload test *to evaluate their basic physical work capacity. Then, based on the results of this test, a clinician used the design utility to create customized workload patterns by adjusting the fuzzy rules for each subject. The subjects then downloaded the exercise program and the latest workload pattern from the central server and performed the *workload control exercise *wearing the unit. The *workload control exercises *by the subjects and the design of the appropriate workload patterns by the clinician were repeatedly performed after the *progressively increasing workload test*. It should be noted that we provided a web-based user interface to assist the users in obtaining the exercise programs [17].

Before every exercise session, we downloaded approximately 450-KB of exercise program data as well as 5-KB of workload patterns from the central server to the exercise area. After every session, we uploaded about 8-MB of measured data, including HR and EMG signals, to the central server and stored it in the database.

**Figure 5:**
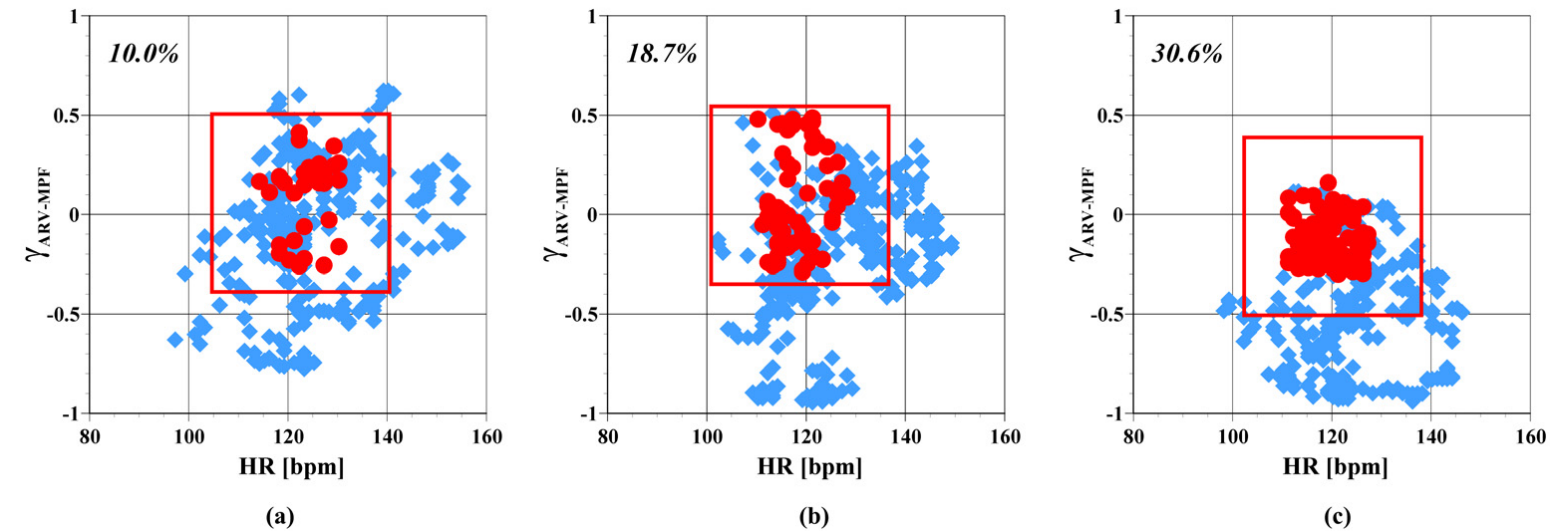
Change in scatter graph between HR and γ_ARV-MPF _for a 22-year-old man during customized exercise session. Exercise (c) is the most effective of the three exercise sessions.

**Figure 6:**
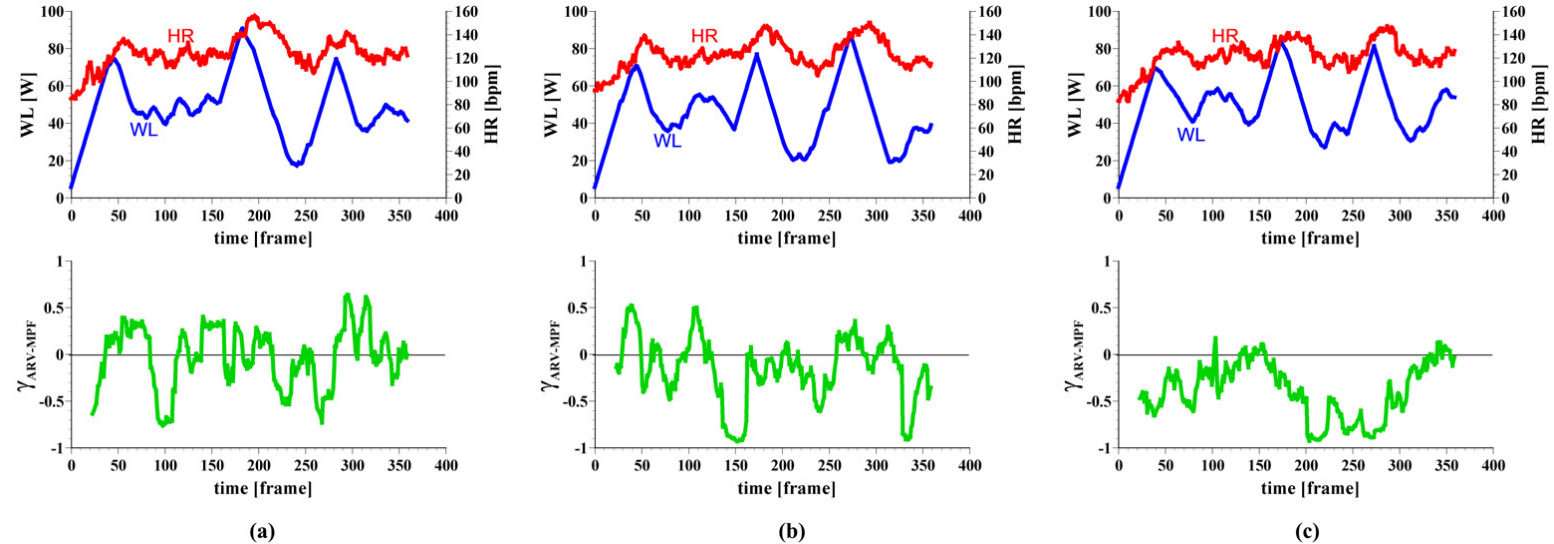
Time-series graphs of different workload patterns for the subject shown in Fig. 5. On the time axis, one frame equals 5 seconds. From top to bottom, workload, heart rate, and γ_ARV-MPF._

Figure 5 shows three HR-γ_ARV-MPF _scatter graphs, ordered by the exercise date. These represent the changes over a 30-minute time period in a 22-year-old man. A muscular fatigue related index, γ_ARV-MPF_, is the correlation coefficient between the averaged rectified value (ARV) and the mean power frequency (MPF) of EMG signals [2], and it became negative as the muscles become fatigued. We also obtained the ratings of perceived exertion (RPE) using Borg's 15-point scale [18] every minute. The RPE is a subjective index widely applied in sports medicine. The exercise levels users found "somewhat hard" are considered efficient based on previous reports. The red squares in each sub-graph represent time slices users found "somewhat hard". There are more samples denoted within the square in (c) (about 30.6%) than there are in (a) (about 10.0%) and (b) (about 18.7%). Therefore, the subject performed more appropriate exercise in Fig. 5 (c). Figure 6 shows the one-to-one time-series graphs for the subject described in Fig. 5. The workload change in (c) was more moderate than it was in (a) and (b). Besides, the maximum workload in (b) and (c) is smaller than in (a). The subject also reported that the workload control pattern shown in Fig. 6 (c), which was designed by reviewing the results of previous exercises, was sufficient to achieve satisfactory exercise. Seven of the nine subjects believed that the workload patterns were challenging at first, but became easier over time. The results of their HRs and EMGs agree with their subjective evaluations. Two male subjects did not obtain satisfying results, but they felt that continuously changing the workload patterns was interesting. The overall results showed that an individualized exercise routine was ensured with the wearable unit in the Internet-based cycle ergometer exercise system.

## Discussion

### Wearable Unit for Personally Customized Machine-Based Exercise

Individualized exercise routines are effective for coping with gradual variations in the physical work capacity and for sustaining the motivation to exercise [1]. In machine-based exercise, a practical operation of personal customization is the continuous provision of appropriate workload patterns for users. Thus, when we apply wearable technology to machined-based exercise, the design of the wearable unit must be able to provide the corresponding control function allowing the user to conveniently and easily follow the prescribed workload pattern. However, most wearable unit studies only provide continuous monitoring of various biosignals [10-14], which we believe is insufficient to meet current demands.

We have presented a new wearable unit design equipped with both measurement and control functions for machine-based exercise. The wearable unit gathers measures of the HR and EMG activity and outputs control signals to the exercise machines. Therefore, it is possible to provide appropriate workload control based on individual biosignals. Our results show that a prototype of the wearable unit, combined with an Internet-based exercise system, can achieve personal customization of cycle ergometer exercise. In our experiments, an external computer estimated the appropriate workloads using a biosignal-based fuzzy control method. As a result, the wearable unit formed a link between the user, the exercise machines, and the external computer in which the exercise programs were executed. The wearable unit provided wired and wireless communication interfaces that connected to the external computers. Such designs are very useful if the wearable unit alone cannot perform the computing task in real time. Most importantly, the wearable unit can accommodate various types of cycle ergometers with different specifications, which will greatly improve the convenience of exercising in different places.

The personal customization process has been ensured with the wearable unit. In our experiments, the clinician successfully customized exercise protocols for most of the subjects based on reviewing the subjects' history data. However, two subjects did not perform the anticipated exercises. This had no relationship with the design of the wearable unit, but most likely occurred because our biosignal-based workload control method was not suitable for them. After all, there are great individual differences in terms of functional flexibility and physical work capacity [1]. We require further fundamental studies on providing appropriate exercise levels, based on biosignals. Moreover, cycle ergometer exercise might not be the preferred approach for some subjects. In this case, other types of exercise might be more useful to them.

### Information Technology to Support Wearable Units

To continuously support the personally customized workload control without constraints on time and place, the wearable unit must be integrated into an Internet-based support system [1,9,19-21], where the exercise routine or design is provided and the measured data is stored and further processed. By transferring the measured data to a central repository, clinicians can review the exercise history and remotely design appropriate workload patterns at their own convenience. Moreover, complicated computing tasks can be assigned to, and the processed results can be acquired from, external computers over wireless connections.

We showed how a wearable unit could be applied to an Internet-based cycle ergometer exercise system. The wearable unit was able to store small amounts of temporal data, and the completed data was processed in an external computer and then uploaded to the database via the Internet. Additionally, the workload patterns and exercise programs were obtained from a central server. Users could perform the individualized exercise routine at any convenient place. Hence, biosignal-based workload control by a wearable unit and the Internet-based support system is a promising approach for providing appropriate exercise levels that will challenge the user and continuously improve their health.

In fact, if we improve the computing performance of the wearable unit by raising the CPU frequency and the internal memory capacity, the unit will be able to compute exercise levels alone. Accordingly, external computers will become unnecessary for control purpose, thus further improving the convenience of the exercise system. For more flexible designs, a removable storage device, which is now being developed, can be used to increase the storage capacity for exercise data and temporal exercise programs. Such design considerations will be implemented in the next version of the wearable unit.

### Range of Application in Health Promotion and Rehabilitation

We described how to apply the wearable unit for an indoor cycle ergometer exercise. The wearable unit could also be effective for outdoor exercises, without requiring any significant changes. We investigated the possibility of using biosignals to control power-assisted bicycles [22]. That study attempted to prevent muscular fatigue during cycling by changing the ratio of rider-generated torque to additional electric-motor-produced torque, based on an evaluation of the measured biosignals. The control process approach is similar to cycle ergometer exercise. Thus, by 1) providing an exercise program that implements the control method, and 2) developing control commands to set the assistance ratio, the wearable unit can also be used to support power-assisted bicycle exercise.

Our wearable unit design for machine-based exercise is suitable for health promotion and rehabilitation. The personal customization process provides an ideal approach and facilitates achievement through the increased motivation of the users, who find convenient not to have to worry about whether or not their exercises are suitable. The workload patterns are remotely designed with the help of clinicians, not by self-assessment of users. Moreover, using Internet-based exercise systems with just one unit, users will be able to perform appropriate exercises on exercise machines that have different specifications. The health promotion and rehabilitation industries are expected to receive favorably control-function-equipped wearable units that can dynamically control the exercise levels, based on measured biosignals.

The wearable unit also reduces the costs of developing and producing exercise machines because the measurement and control functions are separate from the machine. Moreover, loosely coupling exercise machines and exercise programs enables the programs can easily be upgraded without tampering with the hardware, i.e., the exercise machines [23]. The wearable unit helps implement such designs in a more flexible manner, because exercise programs can 1) be installed in the wearable unit to directly control the exercise machines, or 2) reside in an external computer used to communicate with the wearable unit to remotely transfer control signals. Moreover, by taking advantage of the wearable unit, the requirements of exercise machines for the personally customized workload control decrease for practical use, and as a result, the possibility of finding a suitable exercise machine without location constraints would increase.

## Conclusion

We embedded measurement and control functions into a single wearable unit to personal customizing machine-based exercise. Moreover, we introduced the Internet technology to support the personal customization process without time and place constraints. A wearable unit capable of outputting control signals provides the appropriate exercise levels, based on exercise programs and measured biosignals. Users wearing this unit can take advantage of various exercise programs using a variety of exercise machines. A prototype of the wearable unit measured heart rate and EMG signals and wirelessly transmitted the control commands. By applying this unit to an Internet-based exercise system, we were able to personally customize cycle ergometer exercise. The design of our wearable unit is a progressive step towards establishing a convenient and continuously supported wellness environment. In the future, we will be able to apply these units to outdoor exercises and rehabilitation.
